# Multi-genome comprehensive identification of SSR/SV and development of molecular markers database to serve *Sorghum bicolor* (L.) breeding

**DOI:** 10.1186/s12863-023-01165-y

**Published:** 2023-11-03

**Authors:** Yanlin An, Xiaobo Xia, Huayan Zheng, Shirui Yu, Tingting Jing, Feng Zhang

**Affiliations:** 1 Department of Food Science and Engineering, Moutai Institute, Renhuai, China; 2 College of Plant Protection , Nanjing Agricultural University, Nanjing, 210095 China; 3 State Key Laboratory of Tea Plant Biology and Utilization, Anhui Agricultural University, Hefei, China

**Keywords:** SSR, SV, Molecular markers, Polymorphism, Sorghum breeding

## Abstract

**Background:**

As an important food and cash crop, identification of DNA molecular markers is of great significance for molecular marker-assisted breeding of Sorghum (*Sorghum bicolor* (L.) moench). Although some sorghum-related mutation databases have been published, the special SSR and SV databases still need to be constructed and updated.

**Results:**

In this study, the quality of 18 different sorghum genomes was evaluated, and two genomes were assembled at chromosome level. Through the identification and comparative analysis of SSR loci in these genomes, the distribution characteristics of SSR in the above sorghum genomes were initially revealed. At the same time, five representative reference genomes were selected to identify the structural variation of sorghum. Finally, a convenient SSR/SV database of sorghum was constructed by integrating the above results (http://www.sorghum.top:8079/;http://43.154.129.150:8079/;http://47.106.184.91:8079/). Users can query the information of related sites and primer pairs.

**Conclusions:**

Anyway, our research provides convenience for sorghum researchers and will play an active role in sorghum molecular marker-assisted breeding.

## Background

*Sorghum bicolor* (L.) Moench, as an annual C4 herbaceous plant of the family poaceae, has many advantages such as drought tolerance, salt tolerance, barren tolerance, waterlogging tolerance, and strong photosynthetic ability [1–3]. Because of its strong environmental adaptability and excellent agronomic traits, it is not only used as a model plant for plant breeding research, but also used in industrial fields such as chinese hard liquor making, sugar making and bioenergy, which makes it the fifth important cereal in the world and widely planted in hundreds of countries and regions such as tropical, subtropical and temperate zones [4–6]. Diverse growth environments and widespread farm lines have shaped the nucleic acid diversity of the sorghum population gene pool [7]. Identification of molecular markers and database construction based on the newly released genome and sequencing data will promote the development of sorghum breeding to some extent [8].

Compared with restriction fragment length polymorphism (RFLP), randomly amplified polymorphic DNA (RAPD) and amplified fragment length polymorphism (AFLP), simple sequence repeat (SSR) has the advantages of wide genome distribution, simple operation, high polymorphism and co-dominant inheritance [9]. SSR molecular markers have been widely used in many fields such as genetic map construction, genetic diversity analysis and molecular fingerprint construction [10–14]. For example, Adu et al. [15] used 31 SSR markers to analyze the genetic characteristics and population structure of maize populations, and found that 70 maize germplasm could be classified into 5 clusters; Liu et al. [16] identified a total of 667,980 SSR loci in the whole genome sequence of tea plants, and analyzed the genetic structure of 47 cultivated tea plants by using the developed 96 SSR markers; Wu et al. [17] constructed a genetic map of sorghum with a total map length of 997.5 cM and 118 SSR markers based on 277 F2 individuals; Using 32 nuclear genome SSR markers, Zhang et al. [7] analyzed the genetic diversity of 184 sorghum farm varieties from 12 regions in China, which showed that the differentiation among regions and types of Chinese sorghum landraces varieties was relatively weak.

Furthermore, with the advancement of sequencing technology, structural variation on the genome has gradually attracted the attention of more researchers. Studies have shown that although the number of structural variation (SV) is usually less than other mutation sites, it may still have an important impact on the growth and development of plants [18]. For example, the study by Guan [19] and Zhou et al. [20] in peach trees found that a 1.7 Mb structural variation was closely related to the traits of peach tree fruit. Although there have been many reports on the identification of SNP mutation in sorghum and the construction of database, SSR/SV molecular markers still have important application value in breeding field because of their low development cost and easy accurate verification [21–23].

In 2009, the first sorghum genome sequence BTx623 was successfully assembled and released, and the sequencing results showed that the size of the sorghum genome was about 730 Mb [24]. After that, McCormick et al. [25] improved the quality of this genome by deep resequencing, and the number of annotated genes was 24% more than the original genome. Based on nanopore sequencing technology, Deschamps et al. [26] completed the assembly of RTx430 genome, and its scaffold N50 and sequencing accuracy reached 33.28 Mbps and 99.85%, respectively; Cooper et al. [1] completed the sequencing of sweet sorghum genome Rio, and found that there were high non-synonyms and potential functional loss mutations between it and grain sorghum. Recently, Tao et al. [23] published the first sorghum pan-genome sequence and released the reference genomes of 13 sorghum varieties by combining pan-genomics and comparative genomics. The publication of a large number of sorghum genome and third-generation sequencing data provides a basis for exploring the characteristics of SSR loci and structural variation of sorghum from the population level.

At present, the sorghum SNP database has been published and constantly updated, but the database on SSR and SV variation of sorghum is not perfect and needs to be updated [27, 28]. In this study, we identified and compared the SSR loci of eighteen sorghum genomes, and identified the structural variation loci among different sorghum varieties based on the published three generations of re-sequencing data. By integrating these data, we constructed the first SSR/SV database of sorghum, which will play a role in molecular marker development, molecular assisted breeding and genetic diversity analysis.

## Materials and methods

### Collection of genomic and sequencing data

All genome and Pacbio sequencing data used in this study were collected by China National GeneBank database (https://db.cngb.org, project accession number CNP0001440), SorGSD (http://ngdc.cncb.ac.cn/sorgsd/) and SorghumBase (https://www.sorghumbase.org).

### Assisted genome assembly and quality evaluation

Before SSR identification, we first used ragtag software to assemble the sorghum genome at the non-chromosomal level under the default parameters [29]. Subsequently, the N50 of all sorghum genomes was evaluated by Quast [30]. In order to calculate the LTR assembly index (LAI) of different genomes, we first identified the LTR sequences in sorghum genome by using LTR_harvest and LTR_finder software. In order to calculate the LTR assembly index (LAI) of different genomes, we first identified the LTR sequences in sorghum genomes by using LTR_harvest and LTR_finder software, and then integrated the results by using LTR_retriever software to calculate the LAI value [31, 32].

### Identification of multiple genomic SSRs

In order to identify SSR loci and facilitate subsequent analysis, we used SSRMMD software to detect SSR loci in different sorghum genomes [33]. The detection criteria are: di- or tri-nucleotide repeats ≥ 6 times, tetra-nucleotide repeats ≥ 5 times, penta-, hexa- or hepta-nucleotide repeats ≥ 4 times. Meanwhile, the batch design of primers is based on the “connectorToPrimer3.pl” script built into the software. The design principles are as follows: the primer length is between 20 ~ 22 bp, the GC content is between 40% ~ 60%, the annealing temperature difference between upstream and downstream primers is less than 5 °C, and the length of the amplified product is between 150 ~ 250 bp. In addition, SSRMMD software is also used to detect polymorphic SSR between different genomes.

### Identification of structural variations

To detect structural variation, the reference genome was first indexed using minimap2 software, and then the Pacbio sequences were aligned to the reference genome [34]. Convert sam files generated by alignment into bam files and sort them by using samtools. Subsequently, the bam files were indexed and the detection of structural variants was performed using cuteSV software with default parameters.

### Construction of SSR/SV search web

Different from the traditional web building methods, in this study, we chose a python-based web micro-framework Streamlit similar to R shiny to quickly build an interactive web server [35]. All front-end layout and display pages of are completed by html, bootstrap and aggrid components, while data query and filtering services are completed by pandas at the back-end. At the same time, blastn and seqtk software provide sequence alignment and extraction functions, respectively. The whole website is deployed on Alibaba Cloud Ubuntu Lightweight Server.

## Results

### Quality assessment of multiple sorghum genomes

We first assessed the quality of 18 genomes before identifying SSR loci and structural variations. As shown in Table 1, the N50 index of different genomes is quite different, the lowest is the S369-1 variety, whose N50 value is only 24,030,553 bp, while the highest N50 value of the Rio variety reaches 70,703,592 bp. The number of Contig/Scaffold contained in all sample genomes varied from 126 (BTx642) to 3,526 (PI532566). Based on homologous genome assembly technology, we successfully corrected PI532566 and PI536008 draft genomes to the chromosome level, and the number of Contig/Scaffold decreased from 3,526 to 2,860 to 1,124 and 1,329 respectively. In recent years, more and more studies have used LAI value to evaluate the assembly quality of genome. Among all sorghum genomes, 10 have reached the reference genome level (LAI > 10), and reliable assembly quality provides a solid foundation for identification of SSRs and SVs [31, 36].

**Table 1:** Summary of 20 sorghum genome quality evaluation

Genome sample	Assembly size (Mb)	N50 (bp)	LAI	Contig/Scaffold number
BTx623	677	68,658,214	14.40	860
BTx642	657	64,512,399	13.51	126
Rio	705	70,703,592	10.41	271
RTx430	655	64,573,693	13.97	165
SC187	665	65,552,663	12.95	174
353	711	66,905,817	9.81	1,000
AusTRCF317961	614	51,387,152	8.29	1,700
IS12661	594	56,710,613	12.95	704
IS19953	573	57.260,761	7.98	825
IS3614-3	602	57,067,060	13.44	417
IS8525	543	54,182,663	8.50	374
IS929	554	56,127,562	6.95	588
Ji2731	672	64,965,820	12.62	560
PI525695	447	40,754,216	4.55	1,738
PI532566	567	48,793,355	12.71	3,526
PI532566.Chr	560	56,695,343	12.50	1,124
PI536008	644	60,995,127	9.69	2,860
PI536008.Chr	645	64,943,312	9.40	1,329
R931945-2-2	572	55,386,955	12.21	549
S369-1	524	24,030,553	6.98	2,873

### Identification of SSRs in the sorghum genome

After completing the genome quality assessment, we identified SSR loci based on these 18 chromosome levels in sorghum genome. The results showed that there were significant differences in SSR numbers among several sorghum genomes, ranging from 39,120 (PI532566, the variety with the least SSR numbers) to 64,667 (353, the variety with the most SSR numbers) (Table 2). Similar to the research results in other crops [16, 37], SSR frequency gradually decreased with the increase of repeat unit length. Dinucleotide repeat is the most abundant SSR type in sorghum, accounting for 47.49–57.96% in different genomes.

**Table 2:** Detailed information of SSR loci in different sorghum genomes

Genome	Dinucleotide	Trinucleotide	Tetranucletide	Pentanucletide	Hexanucletide	Heptanucleotide	Density (SSR/Mb)
**BTx623**	25,393	11,982	5,276	3,205	2,650	208	72.0
**BTx642**	25,554	14,438	5,450	3,255	2,719	1,031	79.8
**Rio**	26,501	13,621	5,819	3,228	2,708	793	74.7
**RTx430**	26,360	13,971	5,613	3,259	2,743	651	80.3
**SC187**	25,956	14,062	5,692	3,211	2,698	902	79.0
**353**	34,220	16,848	6,049	3,503	2,770	1,277	91.0
**AusTRCF317961**	21,555	10,517	4,937	2,740	2,220	214	68.7
**IS12661**	19,172	10,767	4,858	3,019	2,398	154	68.0
**IS19953**	27,724	12,557	4,380	3,212	2,741	2,836	93.3
**IS3614-3**	20,183	11,076	5,013	2,978	2,433	178	69.5
**IS8525**	25,610	12,520	4,433	3,312	2,835	2,182	93.7
**IS929**	26,833	11,859	4,573	3,319	2,816	532	90.1
**Ji2731**	35,775	13,782	5,777	3,395	2,712	280	91.8
**PI525695**	23,722	10,141	3,327	2,870	2,244	341	95.4
**PI532566.chr**	19,057	10,186	4,573	2,876	2,270	158	69.9
**PI536008.chr**	19,767	11,025	5,193	2,934	2,389	216	64.4
**R931945-2-2**	19,776	10,684	4,790	2,925	2,410	154	71.2
**S369-1**	27,032	10,041	3,779	2,919	2,438	483	89.1

**Fig. 1:**
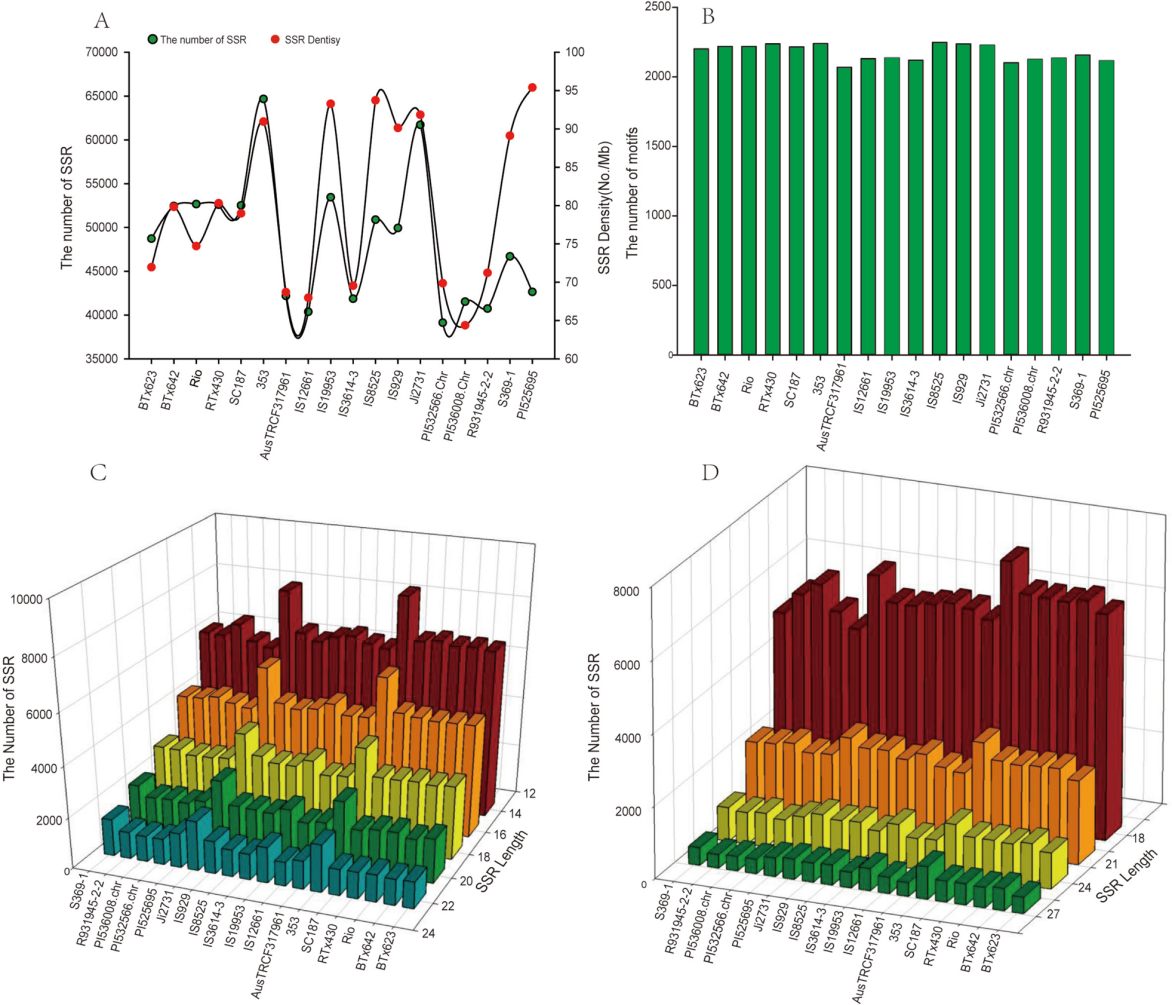
Total number, density, motifs type, number of dinucleotide and trinucleotide SSR in different sorghum genomes. **A** SSR number and density of different sorghum varieties; (**B**) Motif number of SSR in different sorghum varieties; (**C**) and (**D**) represent the number of dinucleotide and trinucleotide SSR in different sorghum varieties, respectively

Among the 18 sorghum genomes, PI525695 has the highest SSR density, reaching 95.4 SSRs/Mb, while the lowest SSR density of PI536008 is only 64.4 SSRs/Mb. The average SSR density of different sorghum genomes was 80.1 SSRs/Mb (Fig. 1A). Different from SSR density, there was little difference in the types of SSR repeat units between different genomes, among which IS8525 had the most repeat unit types, reaching 2,248, while AusTRCF317961 had the least repeat unit type, reaching 2,069. The average number of SSR repeat unit types in the sorghum genome was 2,174 (Fig. 1B). In sorghum genome, dinucleotide and trinucleotide repeat units (SSRs) are dominant (Fig. 1C, D). Especially, the proportion of dinucleotide repeat SSR in different genomes ranges from 44.5 to 58.0%. In addition, for dinucleotide and trinucleotide repeat units, the number of SSR with the length of 12 and 18 bp is the largest, and the number of SSR decreases with the increase of repeat units. In different genomes, the number of dinucleotide SSR and trinucleotide SSR with the length of 12 ~ 22 bp and 18 ~ 27 bp accounted for 61.7%~81.6% and 74.9%~86.6% of the total dinucleotide SSR and trinucleotide SSR, respectively.

**Fig. 2:**
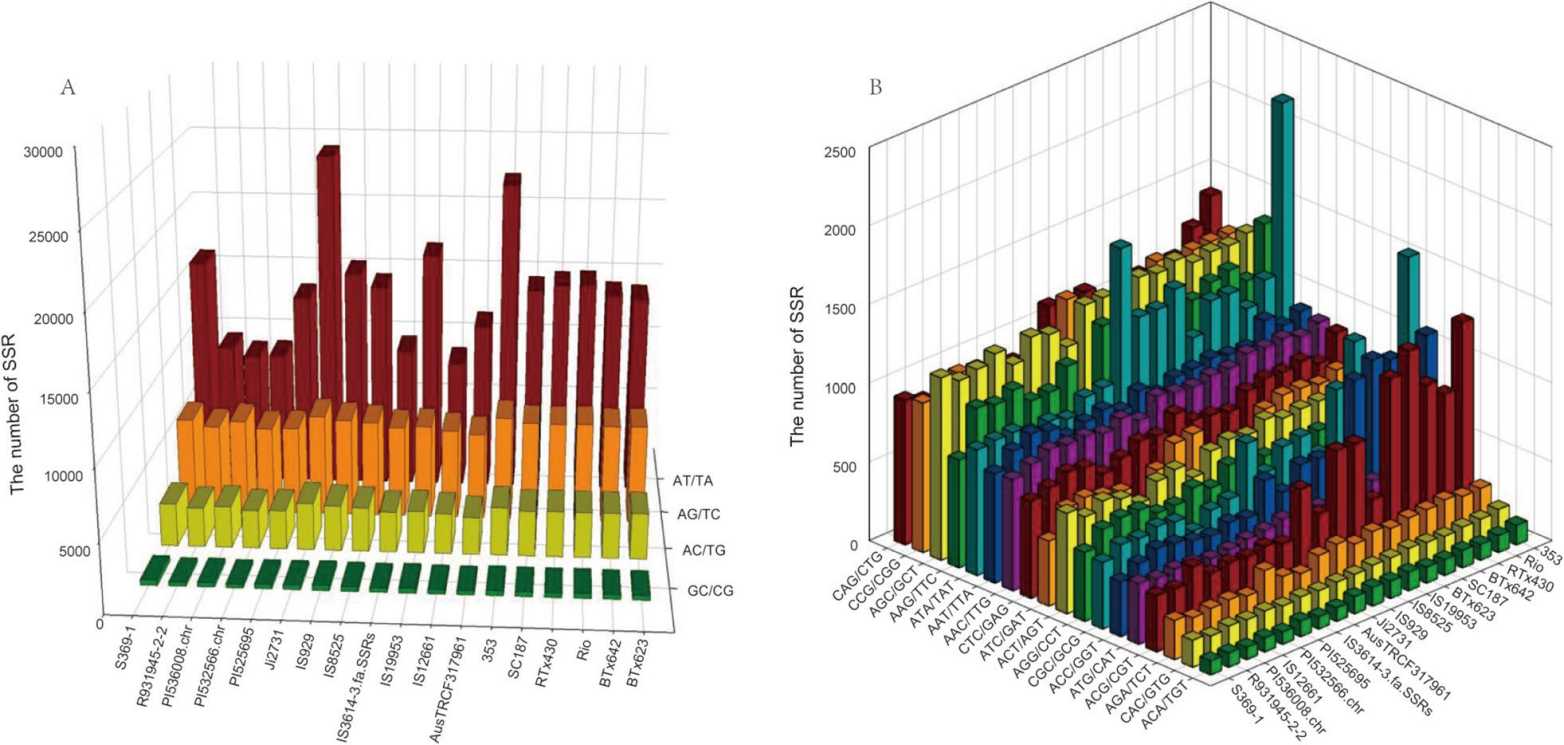
Number of dinucleotide and trinucleotide repeat motifs in sorghum genome. **A** Number of dinucleotide repeat motifs in the genome. **B** Number of trinucleotide repeat motifs in the genome

According to the different motifs, we classify the dinucleotide SSRs into four types: AT/TA, AG/TC, AC/TG and GC/CG, among which AT/TA type SSRs are dominant in different genomes, and the number is in 9,416 (IS12661) and 24,663 (Ji2731) varied, while the number of the least GC/CG type SSRs between different genomes was only 277 (PI532566.chr) to 400 (S369-1) (Fig. 2A). As for trinucleotide SSRs, the types of SSRs that are most abundant in different genomes vary. For example, the number of ATA/TAT in the 353 variety is as high as 2,175, while the number of ATA/TAT in the PI525695 variety is only 750 (Fig. 2B). In the trinucleotide SSR of most samples, more motifs are AAT/TTA, AAG/TTC, CCG/CGG, AGC/GCT and ATA/TAT, while the least two motifs are CAC/GTG and ACA/TGT.

### Identification of structural variations in different sorghum genomes

**Fig. 3:**
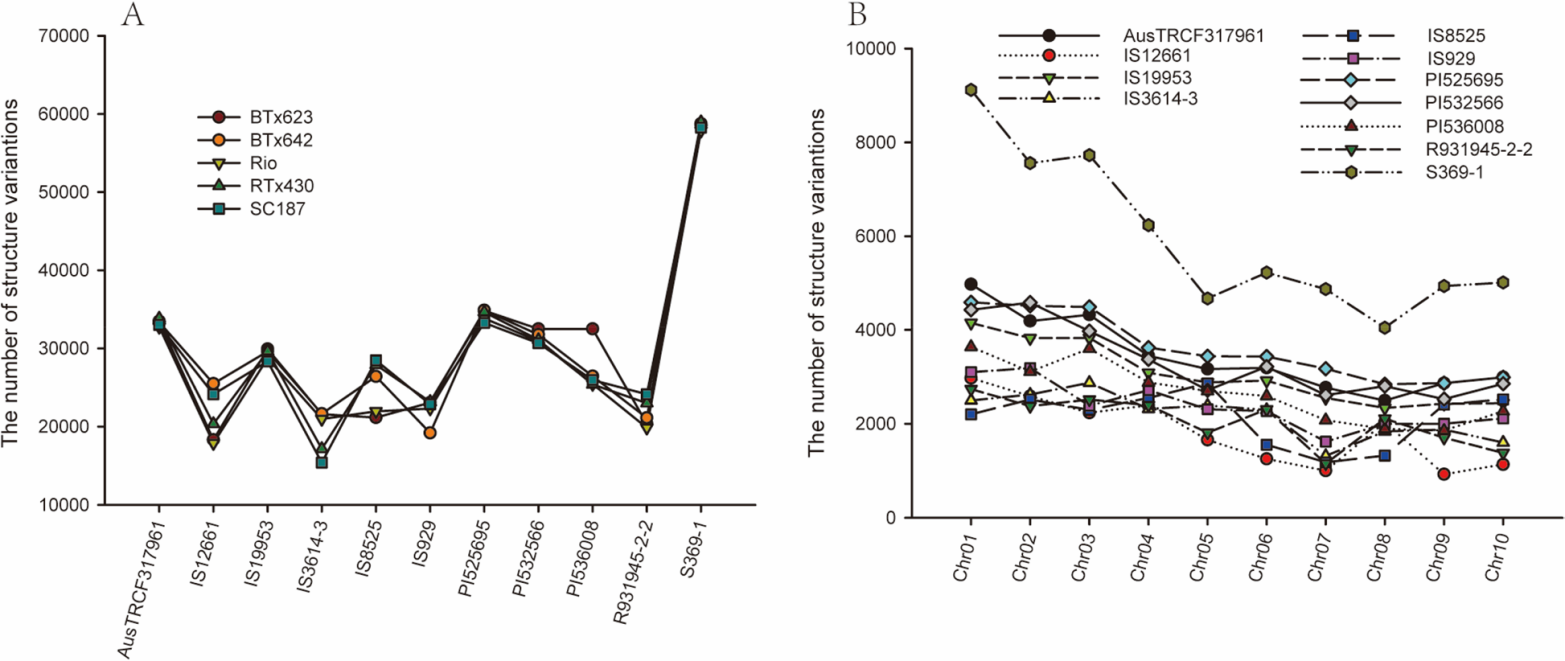
Structural variation of different sorghum genomes. **A** The number of structural variations identified based on five different reference genomes. **B** The number of structural variations on different chromosomes of sorghum

Due to differences in use, growth environment and breeding goals, there may be some genetic differentiation in the genomes of different sorghum germplasms. In order to identify the structural variations in these sorghum genome, we selected five representative sorghum genomes as reference genomes (BTx623, BTx642, Rio, RTx430, SC187) to identify the structural variation of eleven sorghum varieties respectively. As shown in Fig. 3A, the same sequencing data was compared to different reference genomes, and there was little difference in the number of SV variants identified. For example, the number of structural variations identified between AusTRCF317961 and five reference genomes ranged from 32,731 to 33,813. However, there were differences in the number of structural variants between samples. In particular, the number of structural variants between S369-1 and the five reference genomes ranged from 57,857 to 58,968, which was much higher than the other samples. Meanwhile, as shown in Fig. 3B (BTx623 was used as the reference genome), the number of structural variations on sorghum chromosome 1 ~ 10 showed a decreasing trend and was positively correlated with chromosome length.

### Overview of SSR/SV web database and usage cases

**Fig. 4:**
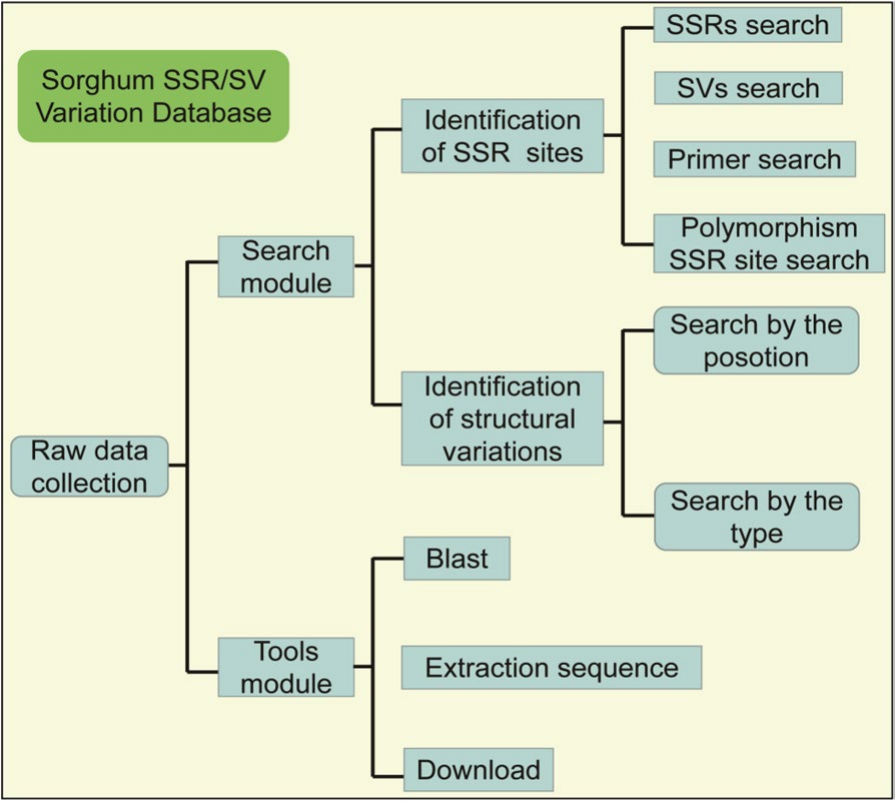
Brief workflow of SSR/SV database construction of sorghum

Based on the above data, we took multiple steps to integrate and construct the first SSR and SV variation database in sorghum (Fig. 4). The whole web page is divided into a sidebar and a display area. In the sidebar, there are two main modules: Search and Tools. The Search module includes four parts: SSR search, Primer search, SV search and Polymorphic SSR site search. Through these functions, users can easily query the variation site information of different samples and obtain suitable amplification primer pairs. Blast, sequence extraction and downloading functions based on different sorghum genomes are provided in Tools module. Once an interactive function located in the sidebar is selected, the corresponding result will be immediately displayed in the main display area (http://www.sorghum.top:8079/; http://43.154.129.150:8079/; http://47.106.184.91:8079/).

**Fig. 5:**
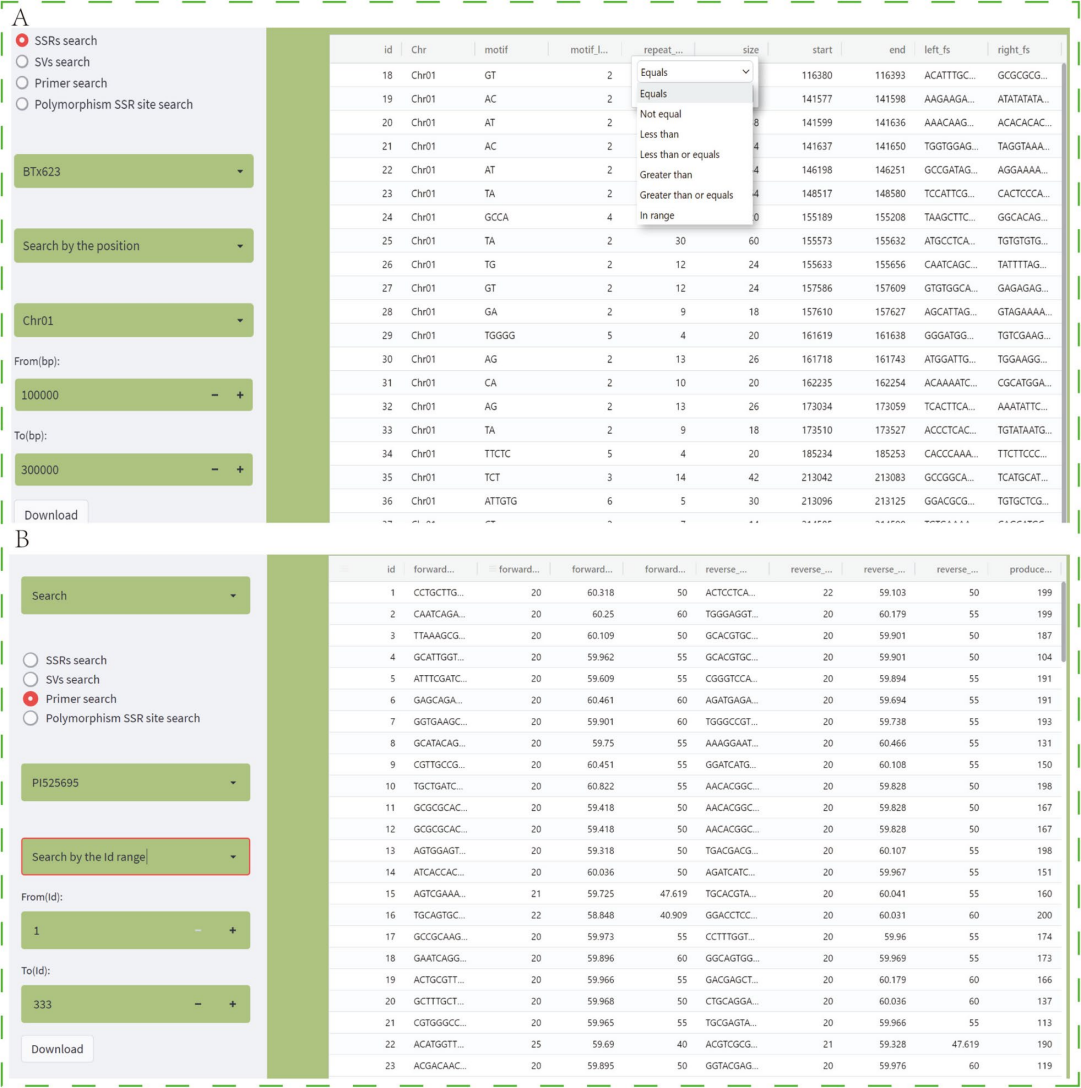
Search tools and examples of results. **A** and (**B**) show SSR and primer search results, respectively

Case study 1, when entering the search module to query SSR, a sample name should be selected first, and then the user can further choose to search by chromosome position or by SSR length. As shown in Fig. 5A, according to the position information, the SSR loci in the range of 100,000 bp to 300,000 bp on chromosome 1 of the BTx623 sample were searched and displayed. In particular, users can also filter each column by conditions according to the search results to obtain the final data set. Then click the Download button to get the data. In order to further obtain primer information of different SSR sites, researchers can select the primer search function to query the corresponding primer pairs according to the SSR id obtained in the previous step. When searching by id range is selected, SSR primer information can be obtained in batches (Fig. 5B). Sometimes, researchers may develop molecular markers for specific samples or genomic regions. In this web site, we provide the search function of polymorphic SSR. Users can select any two different samples to query the polymorphic SSR sites between them, which will improve the development efficiency of molecular markers. In addition, similar to the SSR search function, based on five representative sorghum reference genomes, users can use the SV search function to quickly query the structural variation of different samples.

**Fig. 6:**
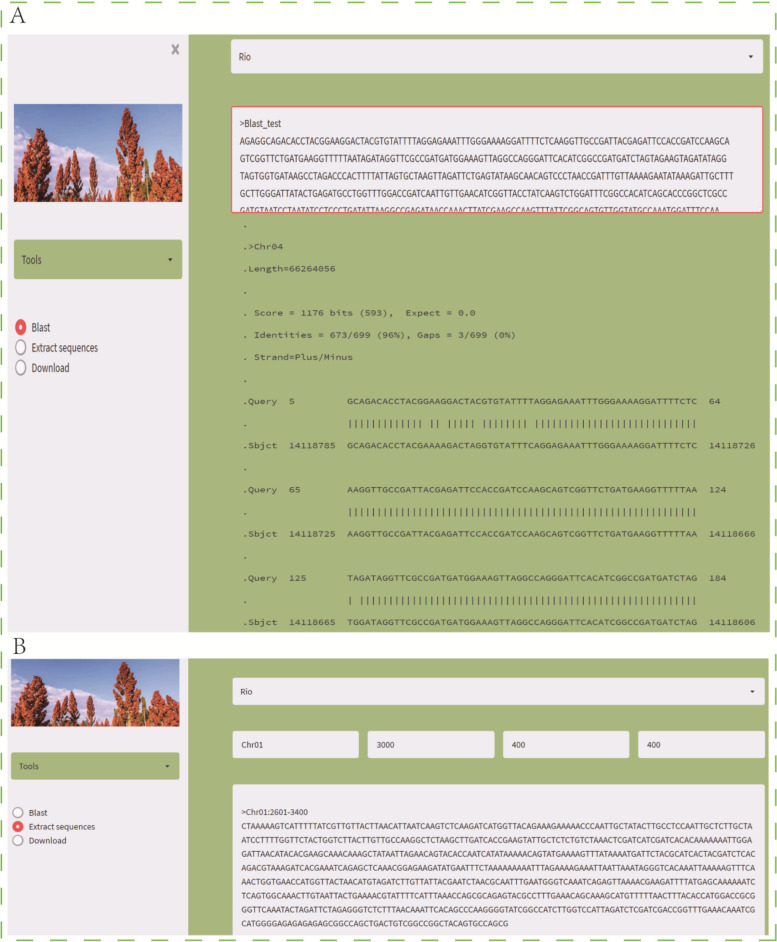
Examples of blast and sequence extraction tools. **A** and **B** show the results of blast and sequence extraction respectively

Case study 2, given that many sorghum genome and pan-genome data have been published, researchers often need to align DNA sequences based on different genomes, so sequence alignment is provided in the Tools module. As shown in Fig. 6A, after selecting the reference genome, enter the DNA sequence with fasta format in the input box, and the alignment result will be displayed in the display area immediately. Sometimes, when researchers need to amplify longer sequences including SSR/SV loci by PCR technology, they can use the “Extract Sequence” function of the Tools module to extract sequences for primer design. As shown in Fig. 6B, the sequences of 400 bp before and after 3000 bp of chromosome 1 of the Rio genome were extracted. After the sequence extraction is completed, the primers can be designed quickly through the quick link part located in the footer of this website.

## Discussion

As the fifth important cereal in the world, the demand of sorghum has been increasing in recent years. In 2021 alone, China’s sorghum imports exceeded 9.4 million tons. However, unlike the sharp increase in demand, the planting area has not been significantly improved, and its molecular breeding process needs to be continuously improved and strengthened. In 2002, Sharopova [38] developed 1,051 SSR markers of maize and used them to construct genetic map; Liu et al. [16, 39] have developed a large number of SSR molecular markers based on the tea plant genome, analyzed the genetic diversity of different tea plant varieties, and constructed unique fingerprints for these tea plant varieties.

In this study, we first evaluated and compared the assembly level of 20 sorghum genomes by N50 and LTR assembly index (LAI). The results showed that the assembly quality of 10 sorghum genomes reached the reference genome level [40]. Although genome-assisted assembly technology can anchor the sequence on the chromosome, thus increasing the length of N50 and reducing the number of contig and scaffold, it has no obvious effect on the increase of LAI value (Table 1). Finally, the SSR loci of 18 chromosome-level sorghum genomes (including two sorghum varieties elevated to the chromosome level by assisted assembly technology: PI532566.chr, PI536008.chr) were identified. The results showed that there were significant differences in SSR numbers among different sorghum varieties, ranging from 39,120 (PI532566) to 64,667 (353), with an average of 48,596.8. While, the SSR density varied from 64.4 (PI536008.chr) to 95.4 SSR/Mb (PI525695). Except for different sequencing varieties, these differences may be related to the size and quality of genome assembly. Similar to previous studies of sorghum and other plants, dinucleotide and trinucleotide repeat units have the most abundant SSR, accounting for 74.2–80.3% in different sorghum genomes [37, 41]. With the increase of repeat unit length, the number of SSR decreased rapidly. Especially, the most abundant motif of dinucleotide repeats in tea plant is AG/CT, while the most abundant motif of dinucleotide repeats in sorghum is AT/TA [16]. In addition, the sequence analysis showed that there were considerable differences in the number of polymorphic SSR among different varieties, ranging from 1,213 (between varieties PI532566 and S369-1) to 17,986 (between varieties SC187 and RTx430), with an average number of 10902.5. The difference of SSR number may be affected by the quality and size of genome assembly and SSR identification methods used in this study.

The application of the third generation sequencing technology has promoted the discovery of plant structural variation and the research progress of functional genes. For example, Yang et al. identified 80,614 polymorphic structural variations by resequencing 521 maize germplasm [18]; Guo et al. [42] research revealed that structural variation plays an important role in regulating the formation of cabbage morphology; An et al. [43] found that a 184 bp structural variation downstream of OVATE gene can regulate the development of tea leaves. In order to promote the discovery and utilization of SV variation in sorghum, we identified the structural variations of sorghum based on five representative reference genomes (BTx623, BTx642, Rio, RTx430 and SC187). The results showed that there was little difference in the number of structural variations identified based on different reference genomes for the same sample. At the same time, the number of structural variations from chromosome 1 to chromosome 10 showed a downward trend in general. Interestingly, S369-1 had more structural variation than the other samples, but the genetic mechanisms responsible for this result need to be further explored.

Effective mining and utilization of large-scale sequencing data plays an important role in promoting plant breeding [22]. Many industrial crops, including cotton [44] and maize [45], have established diversified online databases [46]. For example, Clark et al. [47] identified 369,911 alternative splicing events from 27 tomato project data and built an online database; Liu et al. [48] constructed a comprehensive database of pepper omics and Dubey et al. [49] collected the multi-omics data of tea plants and developed the first SSR database of tea trees. At present, many sorghum genome and sequencing data have been published, but its SSR molecular marker database has not been updated, so we identified and constructed the SSR and SV variation information database based on the published genome and resequencing data to promote the development of sorghum molecular markers and the breeding of high-quality industrial varieties. Through this database, users can quickly obtain SSR and SV variation information of different sorghum varieties, and obtain and redesign specific primers. In order to improve the success rate of marker development, users can further filter the candidate sites by the search function of polymorphic sites. Furthermore, we have established an index for all genomes, and users can enter the Tools module to compare and extract sequences. At the same time, all the result data can be Downloaded through the “Download” function. In a word, the successful publication of this database will promote the molecular marker-assisted breeding of sorghum.

## Conclusions

In this study, we first collected 18 sorghum genomes and preliminarily evaluated their quality. Subsequently, SSR sites in different sorghum cultivars were identified and compared based on the above genome to reveal the distribution characteristics of SSR in these sorghum genome. Based on the published third-generation sequencing data and five high-quality reference genomes, the SV variation of sorghum was identified and analyzed. By integrating the above data, a SSR/SV database of sorghum was successfully constructed. The publication of these results will help relevant researchers to easily obtain relevant data to promote their research process, and will provide support for the breeding of sorghum.

## Data Availability

All supporting data of this study can be easily obtained through Sorghum SSR and SV Database (http://47.106.184.91:8079/).
